# 

**Published:** 1899-07

**Authors:** 


### CONTENTS:

SELECTIONS.

Article I.—Mercurial Necrosis Resulting from Amalgam Fillings. By J. Y. Tuthill, M. D.......... 97

" II.—Some Suggestions on the Treatment of Pyorrhea Alveolaris. G. Lennox Curtis.......... 119

" III.—A Case of Bronchitis and Pneumonia Caused by the Inhalation of the Filling from a Tooth Broken in Extraction. Charles O'Donovan, M. D.......... 125

" IV.—The Deciduous Teeth and the Sixth Year Molars; Disastrous Results from Neglect and their Treatment.......... 129

COMMUNICATED.

Circular Letter of the Foreign Relations Committee of the National Dental Faculties of America.......... 133

MONTHLY SUMMARY.

Antiseptic Uses of Oxygenated Waters.......... 140

Formaldehyde Gas.......... 141

Extracting Superior Cuspids.......... 141

Orthoform.......... 142

Carbolic Acid Poisoning.......... 142

Creosote and Sunlight.......... 143

To Remove Gum Tissue from a Tooth Cavity.......... 143

Bear This in Mind.......... 143

Tannoform Cement.......... 143

Cold Abscesses.......... 143

To Replace a Broken Tooth.......... 144

